# SARS-CoV-2 variants: Impact on biological and clinical outcome

**DOI:** 10.3389/fmed.2022.995960

**Published:** 2022-11-10

**Authors:** Shakuntala Mahilkar, Sachee Agrawal, Sakshi Chaudhary, Swapneil Parikh, Subash C. Sonkar, Dileep Kumar Verma, Vidushi Chitalia, Divya Mehta, Bidhan Chandra Koner, Neetu Vijay, Jayanthi Shastri, Sujatha Sunil

**Affiliations:** ^1^Vector-Borne Diseases Group, International Centre for Genetic Engineering and Biotechnology (ICGEB), New Delhi, India; ^2^Department of Microbiology, Topiwala National Medical College (TNMC) and Bai Yamunabai Laxman Nair (BYL) Charitable Hospital, Mumbai, Maharashtra, India; ^3^Molecular Diagnostic Reference Laboratory, Kasturba Hospital for Infectious Diseases, Mumbai, Maharashtra, India; ^4^Multidisciplinary Research Unit, Maulana Azad Medical College and Associated Hospital, New Delhi, India; ^5^Delhi School of Public Health, Institute of Eminence, University of Delhi, New Delhi, India; ^6^Department of Biochemistry, Maulana Azad Medical College and Associated Hospital, New Delhi, India; ^7^Department of Health Research, Ministry of Health and Family Welfare, New Delhi, India

**Keywords:** COVID-19, SARS-CoV-2, variants, host response, virus evolution, diagnostics, immune escape, coronavirus

## Abstract

The severe acute respiratory syndrome coronavirus-2 (SARS-CoV-2) that was first identified in December 2019, in Wuhan, China was found to be the etiological agent for a novel respiratory infection that led to a Coronavirus Induced Disease named COVID-19. The disease spread to pandemic magnitudes within a few weeks and since then we have been dealing with several waves across the world, due to the emergence of variants and novel mutations in this RNA virus. A direct outcome of these variants apart from the spike of cases is the diverse disease presentation and difficulty in employing effective diagnostic tools apart from confusing disease outcomes. Transmissibility rates of the variants, host response, and virus evolution are some of the features found to impact COVID-19 disease management. In this review, we will discuss the emerging variants of SARS-CoV-2, notable mutations in the viral genome, the possible impact of these mutations on detection, disease presentation, and management as well as the recent findings in the mechanisms that underlie virus-host interaction. Our aim is to invigorate a scientific debate on how pathogenic potential of the new pandemic viral strains contributes toward development in the field of virology in general and COVID-19 disease in particular.

## Introduction

**Figure 1:**
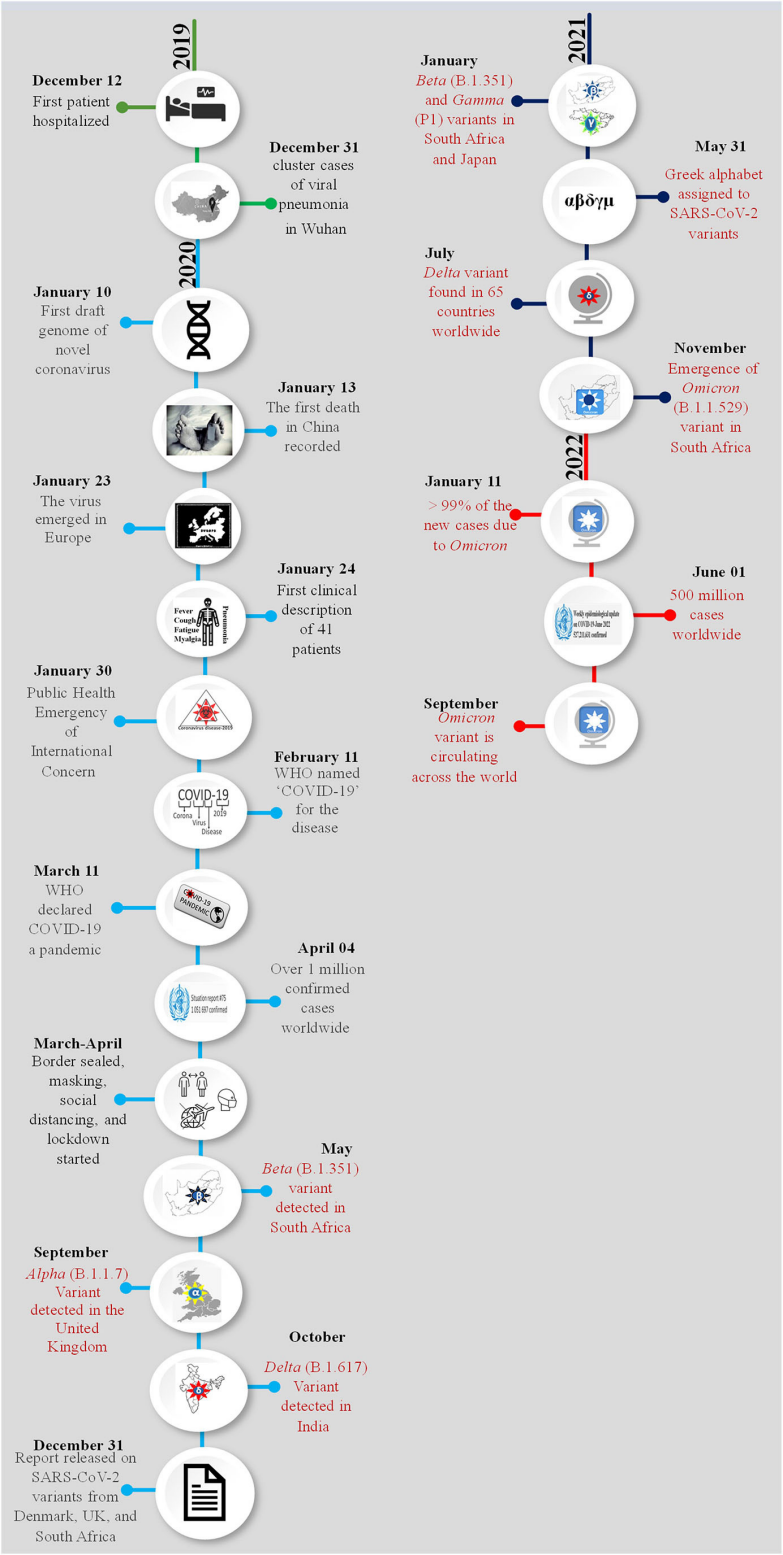
Chronology of the emergence of SARS-CoV-2 variants during the pandemic. The month and date of the main events of the COVID-19 pandemic are presented year-wise and each year is shown with a different color. Appearance of a new variant and related information are highlighted with red color. A pictorial representation of each event has been added in the circle of respective date and month.

Coronavirus induced disease-2019 (COVID-19) initiated a worldwide pandemic since its emergence in December 2019. The disease first appeared as a novel human pneumonia case in Wuhan City, Hubei province, China. Chinese health authorities informed the World Health Organization (WHO) Country Office in China about a cluster of cases by a novel pneumonia-like virus on December 31, 2019 (1). Within a few weeks of its appearance, the disease was identified to be caused by a novel coronavirus and on January 10, 2020, the first draft genome sequence of the new coronavirus was made publicly available *via* a blog post on GenBank (Accession number MN988668) and was named 2019-nCoV (2). On January 24, 2020, a description of the disease from 41 patients was documented (3). Common symptoms associated with the onset of the disease were cough, fever, fatigue, and myalgia. All the identified 41 patients developed pneumonia, 13 out of 41 patients required treatment in the intensive care unit (ICU) of which six patients did not survive, 26/41 patients developed lymphopenia. Patients admitted to the ICU had increased levels of cytokines and chemokines in the plasma (3). Based on the clinical features, the virus was later recognized as the severe acute respiratory syndrome coronavirus two (SARS-CoV-2) and the third *Betacoronavirus* to have caused an outbreak in humans in this century. It was speculated as well as estimated that the SARS-CoV2 had been transmitting from human-to-human since the middle of December 2019 and the transmission was from person-to-person (3–5). After evaluating 425 laboratory-confirmed cases in Wuhan, the mean incubation period of the virus was estimated to be 5 days (6). On January 31, 2020, the WHO announced the disease as a public health emergency of international concern (7). On February 11, 2020, < 2 months after the disease appeared, the disease was named “COVID-19” (8) and on March 11, 2020, the WHO declared COVID-19 a global pandemic (9, 10). Since the pandemic began, the virus has been circulating in the human population and constantly evolving due to mutations and recombination events within its genome. These mutations and recombination events have resulted in the emergence of mutant virus populations and are termed as variants. Early events of the SARS-CoV-2 pandemic and emergence of the variants are shown in Figure 1. These emerging SARS-CoV-2 variants comprising of protein-specific mutations have influenced the epidemiological and the clinical aspects of the COVID-19 pandemic. Specific variants are having enhanced replication efficiency and increased fitness and/or increased transmissibility thereby posing risk of re-infection (11, 12), while others have posed challenges to diagnosis, reducing the protection provided by neutralizing monoclonal antibodies and effective vaccination, leading to the virus being able to escape the host immune system (13). Essentially these variants are causing continual outbreaks of COVID-19 with that has been termed as “waves” that vary in their spread, transmissibility efficiency, and duration of occurrence (14–16). So far, we are still unable to break the transmission of SARS-CoV-2 and this virus is constantly threatening human health, causing several deaths daily worldwide. These variants, therefore, enable SARS-CoV-2 to maintain and/or increase its reproductive fitness and continue to spread even with rising population immunity. In this broad review, we build a schema to understand SARS-CoV-2 variants and their mutations by describing fundamental features of SARS-CoV-2 evolution. We further explain the epidemiological and clinical characteristics of these variants and their associated mutations in disease presentation and management.

## SARS-CoV-2 molecular evolution

**Figure 2:**
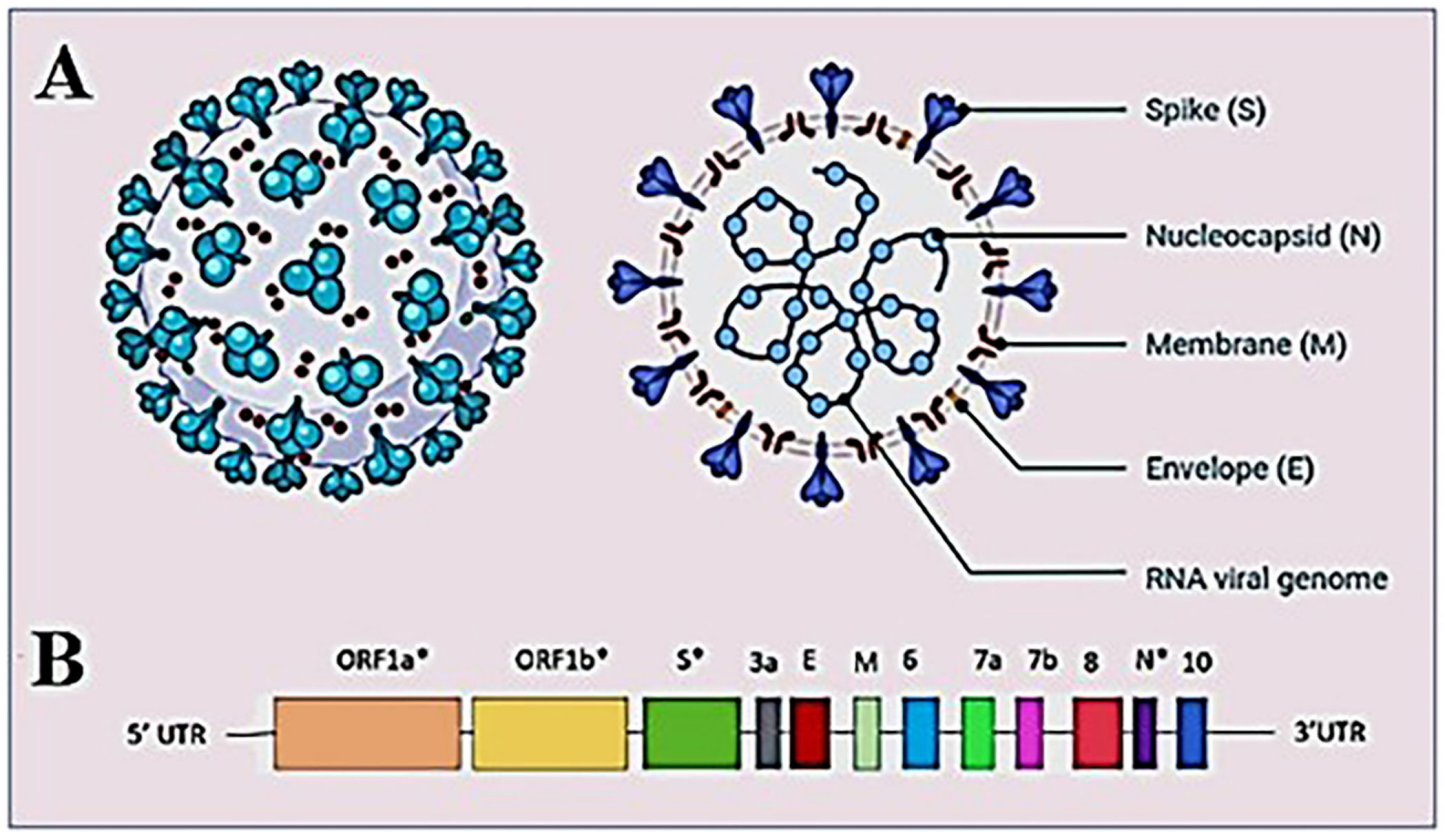
Structure of SARS-CoV-2 virus **(A)** structure of SARS-CoV-2 virion showing the structural proteins, Spike (S), Membrane (M), and Envelope (E) embedded in the host-derived lipid bilayer and Nucleocapsid (N) interacts with the RNA viral genome present at the core of the brain. **(B)** A schematic overview of the SARS-CoV-2 genome structure. *Showing mutation hotspots across the genome.

Coronaviruses have rapidly evolved during the past six decades and are often associated with enteric or respiratory diseases in their hosts. The natural hosts for coronaviruses are mammals and birds and the first human coronavirus was characterized in 1960 by the respiratory tract infection; since then, at least five human coronaviruses have been identified and caused two major outbreaks in the last two decades; the SARS outbreak in 2002 (17) and the MERS outbreak in 2012 (18). Coronaviruses are classified under the order Nidovirales, family *Coronaviridae*, as a member of subfamily *Coronavirinae*. The subfamily is further divided into four genera *Alphacoronavirus, Betacoronavirus, Gammacoronavirus*, and *Deltacoronavirus*. *Alpha* and *Betacoronaviruses* are known to infect mammals while *Gamma* and *Deltacoronaviruses* infect birds. The virus isolated from the patients of the outbreak first detected in Wuhan, China reveals that virus had the typical crown-like structure (19) (Figure 2A). SARS-CoV-2 is a novel β*-coronavirus* consisting of a non-segmented large positive-sense single stranded RNA of 29.9 kb length. The SARS-CoV-2 genome starts from a 5′-cap structure followed by a 5′ UTR subsequently ORF1a/b that encodes 16 nsps that are involved in replication, four genes-encoding structural proteins that include S, E, M, and N proteins followed by six accessory genes encoding six accessory proteins, namely ORF3a, ORF6, ORF7a, ORF7b, ORF8, and ORF10, as well as a 3′ UTR and a poly A tail at the end of the genome (20) (Figure 2B). As of September 15, 2022, 5,504,911 complete sequences are publicly available. The generation of the enormous quantity of genomic data on SARS-CoV-2 has resulted in the need to develop new databases and software to manage the information produced. Several open-access repositories have been developed that play a vital role in the monitoring of SARS-CoV-2's evolution and variations. The most accepted and widely used systems are GISAID (global initiative on sharing avian flu data), Nextstrain, and Pango or Pangolin (Phylogenetic Assignment of Named Global Outbreak Lineages) (21). While GISAID is a rapid and open-access data sharing platform for the viruses having the potential to cause a pandemic such as H5N1 influenza viruses and SARS-CoV-2 (22), Pangolin is a computational tool that assigns the most likely lineage to a given SARS-CoV-2 genomic sequence adhering to the Pangolin dynamic lineage nomenclature scheme (23). Similarly, Nextstrain is a viral genome database that is comprised of data curation and analysis, as well as visualization components (24). Together, these tools help to frame a real-time view into the evolutionary aspects and the range of spread of SARS-CoV-2 and have further expanded to include other viral pathogens that are of high public health importance.

## SARS-CoV-2 mutations and variants

**Figure 3:**
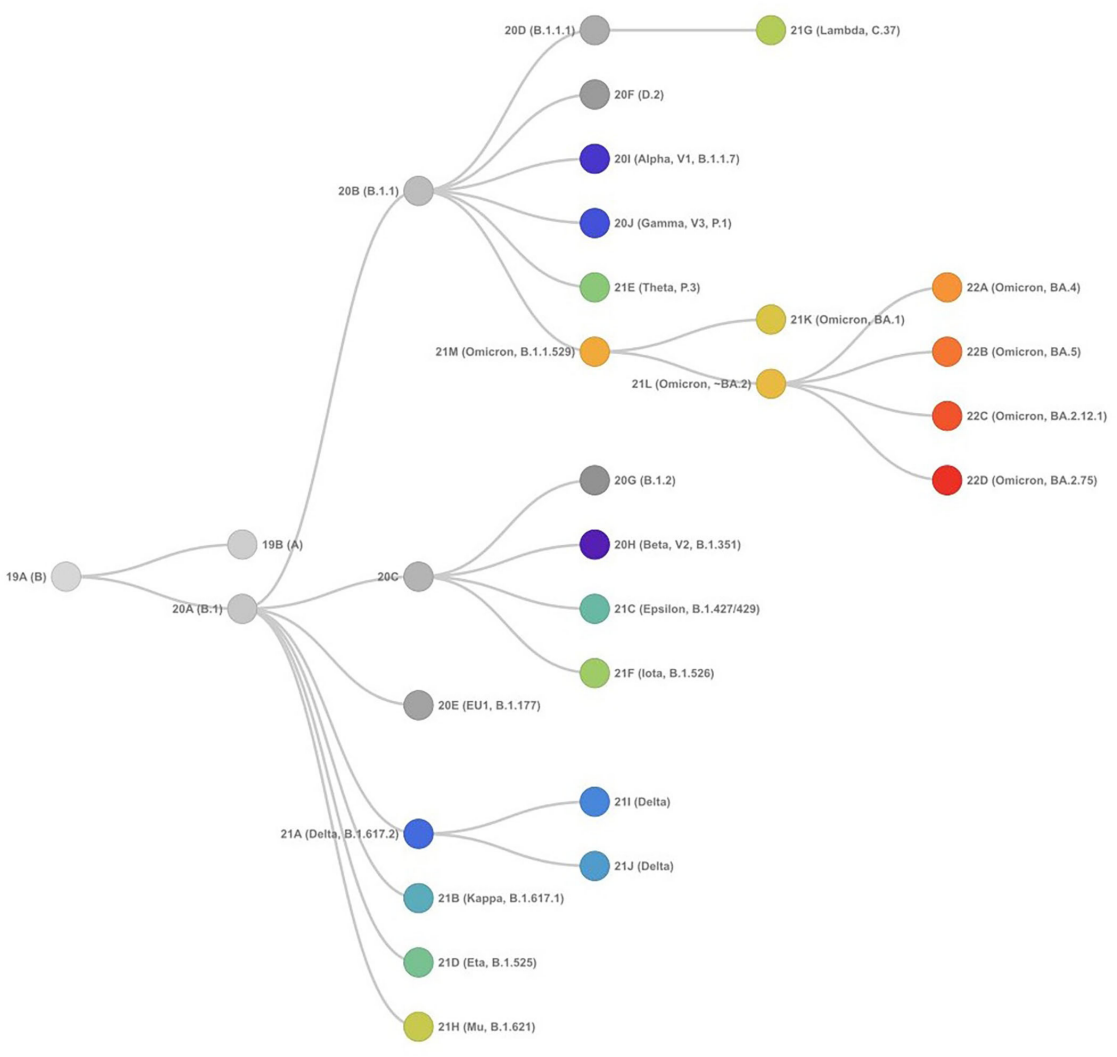
Phylogenetic relationships of SARS-CoV-2 clades, as defined by Nextstrain (as of September 15, 2022) showing evolutionary relationships of SARS-CoV-2 viruses from the ongoing COVID-19 pandemic. The phylogeny is rooted relative to early samples from Wuhan.

SARS-CoV-2 has proved to be a rapidly evolving virus with a high rate of lineage turnover in spite of the proofreading capacity of its RdRp (25–28). This could be attributed to the high transmissibility efficiency of the virus resulting in excessive accumulation of mutations during inter- and intra-host viral replication. The occurrence of mutations and recombination events has resulted in diverse viral populations with their own unique characteristics in disease presentation and transmission. While a mutation occurs spontaneously during the replication process due to low proofreading anility, the mutations that improve viral fitness are further selected (25), a recombination in the viral genome happens when more than one strain co-infect the same host cell and the viral RdRp discontinues the transcription process on one genome and switches to transcribe the other genome resulting in a hybrid genome (29–32). All the above scenarios result in creating a viral species different from the parent virus with its own characteristics and are termed as variants. A group of variants with similar mutations derived from the common ancestor is termed as the lineage of SARS-CoV-2. The SARS-CoV-2 initially delineated into two lineages, i.e., lineage A and lineage B at the root of the phylogenetic tree (23, 33). Lineage A can be defined by the Wuhan/WH04/2020 sequence and appears to share two nucleotides (positions 8,782 in ORF1ab and 28,144 in ORF8) with the closest known bat viruses (RaTG13 and RmYN02), while different nucleotides are present at those sites in viruses assigned to lineage B, which is represented by the Wuhan-Hu-1 strain (19, 34, 35). More information related to SARS-CoV-2 lineages is available at the Pangolin database. The phylogenetic relationship of the SARS-CoV-2 variants forming different lineages rooted from the early samples from Wuhan is presented in Figure 3.

As the virus spread across the globe, it accumulated several mutations that could be analyzed in almost real time owing to the advanced next generation sequencing technologies resulting in a better understanding of the evolution and emergence of variants. Furthermore, the analyses of whole genome sequences have become a helpful tool to study the phylogenetic relationships of viruses, evolutionary rates and the role of mutations in infection and disease severity, transmission patterns, and vaccine development (36). Most importantly, studying these variants has provided important insights into the ongoing epidemiology and evolution of the virus that is being used in surveillance and control of the disease. The World Health Organization (WHO), National Institutes of Health (NIH) and SARS-CoV-2 Interagency Group (SIG), have divided variants into three classes (21).

**Figure 4 (Continued):**
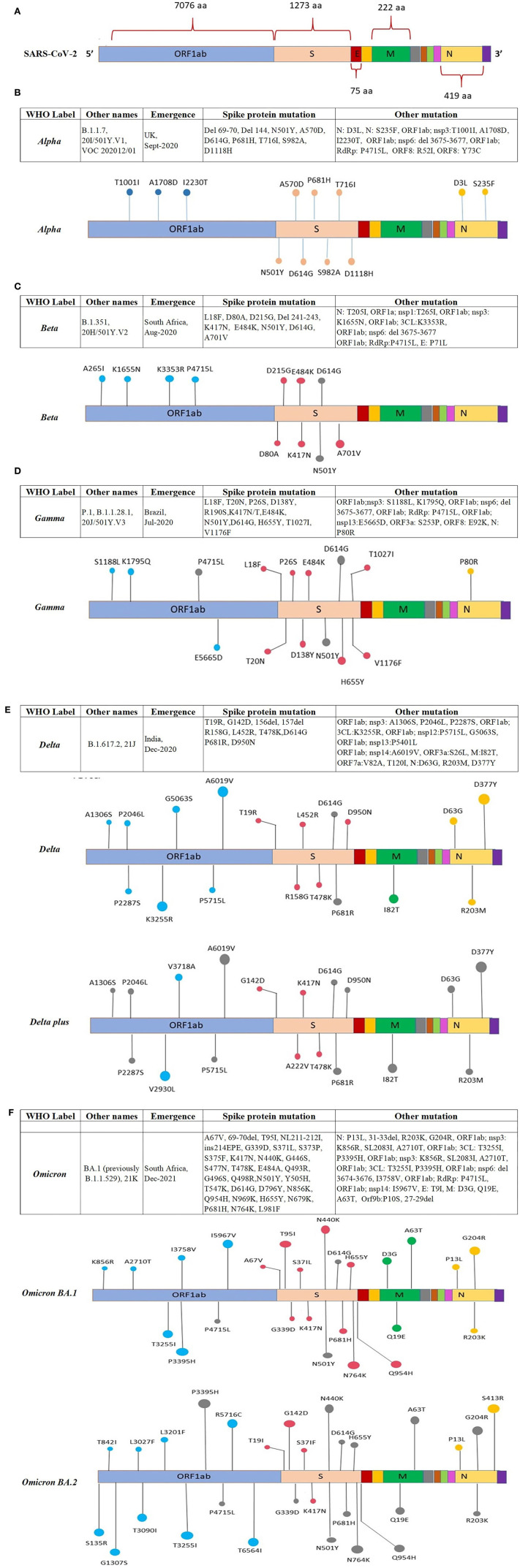
Location of the reported mutations in different regions of the SARS-CoV-2 genome: **(A)** Representing amino acid position of ORFs in the SARS-CoV-2 genome, the ORFs are shown in different colors along with the amino acid position, **(B)** showing mutations in the genome of the *Alpha* variant, **(C)** showing mutations in the genome of the *Beta* variant, **(D)** showing mutations in the genome of the *Gamma* variant. **(E)** Location of mutations reported in various regions of the Delta and Delta-plus SARS-CoV-2 variant. The ORFs of the SARS-CoV-2 genome are represented with different colors and the mutation present on the ORF is indicated on the respective ORFs. **(F)** Location of mutations reported in various regions of the Omicron BA.1 and BA.2 SARS-CoV-2 variant. The ORFs of the SARS-CoV-2 genome are represented with different colors and the mutation present on the ORF is indicated on the respective ORFs.

1. Variants under monitoring (VUM)—Variants designated as VUM include those variants not having clear evidence of phenotypic or epidemiological impact and require constant monitoring and repeated assessment (21). VUM could have additional amino acid changes that are known or suspected to confer the observed change in epidemiology and fitness advantage as compared to other circulating variants. These variants do not cause considerable and imminent risk to public health (Table 1A). VUM is not usually assigned a name until they are converted to variants of interest or variants of concern.
2. Variant of interest (VOI)—A variant having the identified genetic markers that have been linked with altered receptor binding, reduced neutralization by antibodies or vaccination, reduced treatment efficacy, possible diagnostic impact, or predicted enhanced transmissibility or disease severity belongs to this class (Table 1B). *Epsilon* VOI was first detected in March 2020 in California, USA, having five significant mutations: two mutations in the ORF1ab gene, i.e., I4205V and D1183Y, and three mutations in S protein, i.e., S13I, W152C, L452R (37, 38). *Zeta* VOI was first detected in Brazil, having the E484K mutation, but not the N501Y and K417T mutations. *Eta* carried E484K-mutation similar to the *Gamma, Beta, and Zeta* variants, and also holds the ΔH69/ΔV70 deletion similar to *Alpha*, N439K variant, and Y453F variant (39, 40). *Theta* VOI was first reported in January 2021, in the Philippines, it harbors the mutations E484K, N501Y, and other mutations found in other circulating variants (41, 42). *Iota* VOI was first detected in November 2020 in New York City, carrying the two notable mutations S477N mutation and E484K spike mutation (43). The *Kappa* VOI was first identified in October 2021 in India, with three mutations, L452R, E484Q and P681R (44). The *Lambda* VOI was first identified in December 2020, in Peru, South America, carrying two mutations L452Q and F490S in the RBD region (45).
3. Variant of Concern (VOC)—A variant for which there is evidence of increased transmissibility, more severe disease (for example, increased hospitalizations or deaths), a significantly reduced neutralization by antibodies or vaccination, reduced effectiveness of vaccines or treatments, or failures in diagnostic detection (Figures 4A–F).

**Table 1A:** List of variants designated as variants under monitoring according to the WHO as of September 15, 2022.

**Pango lineage**	**GISAID clade**	**Nextstrain clade**	**Relationship to circulating VOC lineages**	**Genetic features**	**Earliest documented samples**
BA.4^#^	GRA	22A	BA.1 and BA.2 sister lineage	BA.2-like constellation in the spike protein + S:del69/70, S:L452R, S:F486V, S:Q493R reversion	South Africa, January-2022
BA.5^#^	GRA	22B	BA.1 and BA.2 sister lineage	BA.2-like constellation in the spike protein + S:del69/70, S:L452R, S:F486V, S:Q493R reversion	South Africa, January-2022
BA.2.12.1	GRA	22C	BA.2 sublineage	BA.2 + S:L452Q, S:S704F	United States of America, December-2021
BA.2.75***	GRA	22D	BA.2 sublineage	BA.2 + S:K147E, S:W152R, S:F157L, S:I210V, S:G257S, S:D339H, S:G446S, S:N460K, S:Q493R reversion	India, May-2022

^#^These lineages have identical constellation of mutations in the spike and the following differences outside the spike: BA.4: ORF7b:L11F, N:P151S, ORF6:D61L, ORF1a:del141/143; BA.5: M:D3N.

***Additional mutation outside the spike protein: ORF1a:S1221L, ORF1a:P1640S, ORF1a:N4060S; ORF1b:G662S; E:T11A.

**Table 1B:** List of variants identified as variants of interest as of September 15, 2022 according to covariant database and the WHO.

**WHO label**	**Other names**	**Emergence**	**Spike mutation**	**Other mutations**
*Epsilon*	B.1.427, California (CA) B.1.429, CAL.20C	USA, September-2020	S13I, W152C, L452R, D614G	ORF1a:T265I, S3158T, I4205V, ORF1b:P314L, (P976L), D1183Y, ORF3a:Q57H, N:T205I
*Zeta*	P.2, B.1.1.28.2	Brazil, October-2020	E484K, F656L, D614G, T859I, V1176F	
*Eta*	B.1.525, 20A/S:484K	Worldwide, December-2020	Q52R, A67V, Del 69-70, Del 144, E484K, D614G, Q677H, F888L	ORF1a:T2007I, ORF1b:P314F, N:D3Y, N:A12G, N:T205I, M:I82T, E:L21F, E:I82T, del:11288:9, del:21765:6, del:28278:3
*Theta*	P.3, B.1.1.28.3, 21E	Philippines, January-2021	E484K, N501Y, D614G, P681H E1092K, H1101Y, V1176F	ORF1ab:L3201P, D3681E, L3930F, P4715L, ORF8: K2Q N:R203K, G204R
*Iota*	B.1.526, 21F	USA, November-2020	L5F, T95I, D253G, S477N, E484K, D614G, A701V	ORF1ab:del3675-3677, ORF1b:P314L, ORF1b:Q1011H, ORF3a:P42L, ORF3a:Q57H, ORF1a:T265I, ORF1a:L3201P, N:P199L, N:M234I, ORF8:T11I
*Kappa*	B.1.617.1, 20A/S:154K	India, October-2021	T95I, G142D, E154K, L452R E484Q, D614G, P681R, Q1071H	ORF1b:P314L, ORF1b:G1129C, ORF1b:M1352I, ORF1b:K2310R, ORF1b:S2312A, N:R203M, N:D377Y, M:I82S, ORF3a:S26L, ORF1a:T1567I, ORF1a:T3646A, ORF7a:V82A
*Lambda*	C.37, B.1.1.1.C37	Peru, December-2020	G75V, T76I, Del 246-252, L452Q, F490S, D614G, T859N	ORF1a:T1246I, ORF1a:P2287S, ORF1a:F2387V, ORF1a:L3201P, ORF1a:T3255I, ORF1a:G3278S, ORF1b:P314L, N:P13L, N:R203K, N:G204R, N:G214C

The *Alpha* VOC was first detected in the UK in September 2020 with a fitness advantage over the original strain and quickly became the predominant variant circulating in the UK (46). The *Alpha* VOC contains 17 mutations in the viral genome, out of which 14 mutations are non-synonymous point mutations and three are deletions. Among these, eight mutations are present in S protein: Δ69–70 deletion, Δ144 deletion, N501Y, A570D, P681H, T716I, S982A, and D1118H (47, 48) (Figure 4B).

The *Beta* VOC was first detected in South Africa in December 2020, and contains nine mutations in the S protein: L18F, D80A, D215G, R246I, K417N, E484K, N501Y, D614G, and A701V, of which three mutations; K417N, E484K, and N501Y are detected in the RBD of the S protein. The N501Y was also identified in the *Alpha* variant (47, 49, 50) (Figure 4C).

The *Gamma* VOC was first detected in Japan in January 2021, probably through travelers returning from Brazil (138) which was found to have evolved from a regional B.1.1.28 lineage in the Amazon in November 2020 and within 2 months predominated the parental lineage (51). The *Gamma* VOC contains 10 mutations in the S protein, i.e., L18F, T20N, P26S, D138Y, R190S, H655Y, T1027I V1176, K417T, E484K, and N501Y (52), of these mutations, L18F, K417N, and E484K are found in the RBD of spike protein as previously reported in the *Beta* VOC (47, 50, 53) (Figure 4D).

The *Delta* VOC was first reported in India in October 2020 and within a few months, it spread to other countries. It was detected with E484Q and L452R S protein mutations. The *Delta* VOC has 13 mutations in the genome; four specific mutations located in spike protein T478K, D614G, L452R, and P681R, are of concern (54–56). The *Delta Plus* variants are classified as sub-variants of the *Delta* variant that are having several important mutations in the spike protein. They are structurally very similar to *Delta* VOC but have a few changes. *Delta Plus* has a K417N mutation in the spike protein. The sub-variants AY.1, AY.2, AY.3, and AY.4 are named *Delta Plus* (57) (Figure 4E).

The *Omicron* VOC was detected in November 2021 in South Africa, containing more than 30 mutations to the S protein, and evolved as a highly divergent variant with more than 60 mutations overall. The *Omicron* VOC was recently divided into five lineages, i.e., BA.1, BA.2, BA.3, BA.4, and BA.5 (58). The BA.1 lineage contains one mutation in the E protein, 37 mutations in the S protein, six in the N protein and two in the M protein. BA.2 lineage contains 57 mutations, of which 31 in the S protein; the N-terminus is considerably different as compared to BA.1 (59). While BA.3 lineage carries a combination of mutations found in the S protein (a total of 33 mutations) of BA.1 and BA.2 lineages. The 31 mutations of the S protein in BA.3 are common to BA.1, and two mutations are common to the BA.2 lineage. BA.3 caused fewer infections as compared to BA.1 and BA.2 probably due to the loss of the S protein mutation present in the BA.1 and BA.2 lineages (60) (Figure 4F). The Omicron BA.4 and BA.5 lineages appeared in April–May 2022 (58, 61). There are a total of four amino acid differences between the BA.4 and BA.5 lineages, in proteins such as OFR1a, ORF6, ORF7b, and Nucleocapsid (N), with one amino acid difference in each protein, while four mutations in the S protein that is different in the BA.4/BA.5 as compared to the BA.2 lineage (58). So far BA.4 and BA.5 have not reported with severe illness, but constant monitoring is required to prevent further spread which may result in another variant with increased fitness that may cause severe illness.

## Impact of SARS-CoV-2 variants on Diagnostics/Detection

The accessibility of the complete genome sequences of SARS-CoV-2 in the beginning of the pandemic facilitated the rapid development of the assays for detection of SARS-CoV-2 using real-time reverse transcriptase Polymerase Chain Reaction (rRT-PCR) (62). The first protocol describing the rRT-PCR assay was published in Europe on January 23, 2020 (48, 62, 63). The report defined specific primer and probe sequences for various SARS-CoV-2 specific targets such as RNA-dependent RNA-polymerase (RdRp) and nucleocapsid (N) genes along with the broad range probe pan-Sarbeco that could detect 2019-nCoV along with SARS-CoV and bat-SARS-related CoVs (63). The recommendations included the screening of the samples using the E gene target followed by confirmation using the RdRp gene target in samples positive for E gene (63, 64). Procedures used for collecting, transporting, and storing the specimens and strict adherence to all the protocols for sample collections has shown to be imperative for correct and valid results (65–68). RT-PCR (qRT-PCR) is established as the clinically acceptable and most widely used test for SARS-CoV-2 detection and is a routine confirmatory test for SARS-CoV-2 as advised by the WHO. Despite the many advantages RT-PCR has in SARS-CoV-2 diagnostics, the testing method is not without its limitations, specifically when it comes to the correct detection of variants of SARS-CoV-2 (69). In a bid to overcome this issue, several point-of-care diagnostic tests were developed for SARS-CoV-2 variant detection (Table 2). Point-of-care tests (POCTs) can not only be performed in clinical laboratories but also can be used by trained non-laboratory personnel in patient care facilities, bringing the diagnostic test for SARS-CoV-2 closer to patients (70–72). A comparison of different gene targets and primers used in nucleic acid amplification methods in lab-based and point-of-care nucleic acid amplification tests of SARS-CoV-2 around the world is shown in Table 2.

**Table 2:** List of different gene targets and primers used in lab-based and point-of-care nucleic acid amplification tests for SARS-CoV-2 around the world.

**Institute**	**Gene target**	**Probe (5^′^-3^′^)**	**Forward primer (5^′^-3^′^)**	**Reverse primer (5^′^-3^′^)**
China CDC	ORF1ab gene	FAM-CCGTCTGCGGTATGT GGAAAGGTTATGG-BHQ1	CCCTGTGGGTTTTACACTTAA	ACGATTGTGCATCAGCTGA
	N gene	FAM-TTGCTGCTGCTTGACAGA TT-TAMRA	GGGGAACTTCTCCTGCTAGAAT	CAGACATTTTGCTCTCAAGCTG
US CDC	N1 target	FAM-ACCCCGCATTAC GTT TGGTGGACC-BHQ1	GAC CCC AAA ATC AGC GAA AT	TCT GGT TAC TGC CAG TTG AAT CTG
	N2 target	FAM-ACAATTTGCCCCCAGCGC TTCAG-BHQ1	TTA CAA ACA TTG GCC GCA AA	GCG CGA CAT TCC GAA GAA'
	N3 target	FAM-AYCACATTGGCACCCGCA ATCCTG-BHQ1	GGG AGC CTT GAA TAC ACC AAA A	TGT AGC ACG ATT GCA GCA TTG
France Pasteur Institute	RdRP1 target	HEX-AGATGTCTTGTGCTGCCG GTA-BHQ1	ATGAGCTTAGTCCTGTTG	CTCCCTTTGTTGTGTTGT
	RdRP2 target	FAM-TCATACAAACCACGCCAG G-BHQ1	GGTAACTGGTATGATTTCG	CTGGTCAAGGTTAATATAGG
Japan National Institute of Infectious Disease.	N gene	FAM-ATGTCGCGCATTGGCATG GA-BHQ	AAATTTTGGGGACCAGGAAC	TGGCAGCTGTGTAGGTCAAC
Germany Charité	RdRP gene	FAM-CAGGTGGAACCTCATCAG GAGATGC-BBQ	GTGARATGGTCATGTGTGGCGG	CARATGTTAAASACACTATTAGCATA
	E gene	FAM-ACACTAGCCATCCTTACTGCGCTTCG-BBQ	ACAGGTACGTTAATAGTTAATAGCGT	ATATTGCAGCAGTACGCACACA
Thailand National Institute of Health	N gene	FAM-CAACTGGCAGTAACCA-BQH1	CGTTTGGTGGACCCTCAGAT-	CCCCACTGCGTTCTCCATT
Hong Kong University	ORF1b-nsp14 gene	FAM-TAGTTGTGATGCWATCATGACTAG-TAMRA	TGGGGYTTTACRGGTAACCT	AACRCGCTTAACAAAGCACTC
	N gene	FAM-GCAAATTGTGCAATTTGCGG-TAMRA	TAATCAGACAAGGAACTGATTA	CGAAGGTGTGACTTCCATG

In India, the initial testing using the kit designed by the Indian Council of Medical Research-National Institute of Virology (ICMR-NIV) was based on the recommended testing strategy with the addition of the human specimen control, *RNaseP* (71, 73). The assay employed the E gene for initial testing with *RNaseP* as a control for efficient sample collection and RNA extraction, followed by testing for SARS-CoV-2 specific RdRp primers and probes. These were multiple-tube uniplex assays to be done in succession. However, it became evident that the SARS-CoV-2 positive samples with low viral load were being missed or marked as presumptive positive due to a comparative lower sensitivity of the RdRp gene, resulting in false negative reporting (74). Owing to this caveat, samples required repeat testing utilizing more time, labor, and reagents. In order to mitigate the possible sensitivity issue, an additional target ORF1b gene was introduced, as the ORF gene offered a higher sensitivity as compared to the RdRp gene (75, 76). The advent of the single-tube multiplex assays for SARS-CoV-2 proved advantageous with respect to time and labor. However, each commercial kit differed in its targets and sensitivities (76–78). Therefore, having multiple confirmatory genes served to compensate for the sensitivity and specificity of its targets.

In addition to the number of genes, gene target regions also played a crucial role. The first indication was in November 2020 with the emergence of the *Alpha* variant where in a widely used commercial kit for the detection of SARS-CoV-2, the S gene failed to amplify; the phenomenon was later termed as the S gene target failure (SGTF). The SGTF was a result of the H67Del/V70Del mutation associated with immune evasion in the *Alpha* variant (48, 79, 80). However, due to the presence of multiple other SARS-CoV-2 specific genes (ORF1ab and N gene), the positive samples could be correctly identified. The emergence of the *Alpha* variant, the first Variant of Concern, brought about a paradigm shift underlining the impact of the genetic variation as a result of the evolution of the virus on its detection. This highlighted the significance of the assessment of genetic variability affecting the sensitivity and specificity of the assays. Therefore, the binding of the primer and probe sequences were assessed for all the subsequently emerging variants. A similar situation arose with the emergence of the *Omicron* variant containing numerous mutations in the S and N genes. But in the case of the *Omicron* variant, the mutations not only affected the diagnostics but also the effectiveness of the treatment (66, 81). Therefore, genomic surveillance proved an effective tool in disease management in addition to the diagnostic surveillance systems. Although whole-genome sequencing (WGS) remains the most accurate approach for genotyping, there is a limited capacity for WGS to be adequate for the surge of cases; also, it is not feasible or sustainable for most laboratories. Additionally, it is cost and labor intensive, requires a skilled person to administer, and has a longer turn-around time (82, 83). Therefore, rapid and accurate molecular tests for differentiating between the variants are the need of the hour. While SGTF was used for the initial screening for the *Omicron* variant, it is not exclusive to the *Omicron* variant as the H67Del/V70Del mutation is shared by the Alpha and other variants (82, 83). Furthermore, target amplification failure is not as reliable a technique as positive identification (82). In order to facilitate the same, various manufacturers have optimized rRT-PCR assays that can differentiate the variants based on specific amplification of the target mutation. Two such kits are approved and available in India, which can differentiate the *Omicron* VOC from the other variants of SARS-CoV-2 (84).

Overall prevention and efficient control strategies strongly depend upon the diagnostic testing for SARS-CoV-2. The WHO recommends Nucleic Acid Amplification Test (NAAT) such as rRT-PCR based on unique sequence of viral genome for diagnosis of SARS-CoV-2. On emergence of newer variants, the uniqueness of the target sequence of viral genome has changed, needing newer targets for primer designing. Hence, with every evolving mutation, the diagnostic landscape changed and will keep on changing. Outside clinical and laboratory settings, antigen detection-based lateral flow immunoassays (LFA) have also been recommended by the WHO. Antigen-detecting rapid diagnostic tests (Ag-RDTs) offered a faster and less expensive way to diagnose an active SARS-CoV-2 infection (85), Ag-RDT can be used outside of clinical and laboratory settings, including communities as a POCT. Similarly, the ICMR also recommended the use of rRT-PCR-based tests as the frontline diagnostic test for SARS-CoV-2 and Ag-RDT assays for the detection of present infection. Serological antibody testing is currently recommended for use only in research settings and for serosurveillance purposes. All diagnostic commodities, namely nucleic acid amplification assays, antigen detection assays (both POCT and laboratory settings based), and serological assays (Antibody detection) approved by the US FDA EUA/ Japan PMDA/ Australia TGA/ WHO EU, are eligible for use under intimation to the Drug Controller General of India (DCGI). All other diagnostics needed to be evaluated and certified by ICMR before their use in the country (86).

Diagnostic challenges are still a concern because of the lack of confirmative and accurate diagnosis of mutations in the terms of ASSURED diagnostics (acronym for Affordable, Sensitive, Specific, User-friendly, Robust and rapid, Equipment-free, and Deliverable to end-users). Regular monitoring and evaluation of the potential impact of genetic variants on the diagnostic assays especially rRT-PCR and Ag-RDT assays is an urgent need. The basic principle, advantages, disadvantages, and turn-around time for each test used for clinical diagnosis and genomic surveillance of SARS-CoV-2 are listed in Table 3. As we know that the virus spreads through air droplets, there is evidence of air-born transmission also, resulting in unique transmission patterns (87–89). However, differential mode of transmission of any variant has not yet been reported.

**Table 3:** Widely used diagnostics approaches for SARS-CoV-2 detection.

**Method**	**Principle**	**Advantage**	**Duration**	**Disadvantage**
Next-generation sequencing (NGS)	Whole-genome sequencing	Highly sensitive and specific provides all related information. Can identify a novel strain	1–2 day	High expertise, Equipment dependency and high-cost Highly sophisticated Lab required
RT-PCR	Specific primer-probe based detection	Fast results, higher sensitivity, need small amount of DNA, can be performed in a single step. Well-established methodology in viral diagnostics	3–4 h	Higher costs due to the use of expensive consumables. Expensive lab equipment. Detection is also complex and time-consuming. May be affected by novel mutations in emerging variants. US FDA listed some molecular assays expected to fail to detect the SARS-CoV-2 omicron variants (https://www.fda.gov).
RT-LAMP	More than two sets of specific primers pair-based detection	Highly repeatable and accurate. Single working temperature	1 h	Too sensitive, highly prone to false positives due to carry-over or cross-contamination
Antigen detection assay	Lateral Flow Immunoassay (LFA)	POCT, Samples does not need to be transported to the laboratory, saving time and cost	15–30 min	Testing comes after 3–4 days of infection. Lower sensitivity and specificity
	CLIA, ELISA	Laboratory setting	4–6 h	Testing comes after 3–4 days of infection. Lower sensitive and specificity
Serological assays	Lateral Flow Immunoassay (LFA	POCT	15–30 min	Detected 1–2 weeks after vaccination or infection based on the type of antibody to be detected
Antibodies (IgG/IgM)	ELISA, CLIA	Laboratory setting	4–6 h	Detected 1–2 weeks after vaccination or infection.
CT scan	Chest images	Enhance sensitivity of detection if findings combined with RT-PCR results	1 h	Indistinguishability from other viral pneumonia and the hysteresis of abnormal CT
Virus culture and isolation	*In vitro* live virus isolation and propagation	Highly (100%) specific gold standard	5–15 days	Low sensitivity as isolation is not 100%

## Impact of SARS-CoV-2 variants on clinical presentation and treatment

SARS-CoV-2 infection may be asymptomatic or may result in symptomatic disease of varying severity with the involvement of multiple organ systems such as the respiratory system, gastrointestinal system, cardiovascular system, nervous system, or multi-system involvement (90). Many factors contribute to the clinical presentation. Host-specific factors such as age, pre-existing high-risk conditions, gender, and pregnancy are known to impact clinical presentation and severity of infection (90). Host immunity from vaccination, a past infection, or both, can significantly impact clinical presentation (91). Pathogen factors can also impact the clinical presentation and severity; variant-specific clinical presentation and severity have been a topic of great interest. Different variants may have specific mutations and amino acid changes in spike and non-spike proteins that can impact the clinical presentation and severity (92). A variant may have increased virulence and may therefore cause more severe disease. However, the appearance of different variants in different geographic areas at different time points has resulted in a scenario of a varied clinical presentation and severity thereby hinting on the role of host immunity in disease presentation. A variant that appears after the vast majority of a population has immunity from vaccination and/or past infection may appear to be “milder” due to host immunity rather than specific viral mutations. Viral mutations may have an impact on clinical presentation due to altered tissue tropism or due to increased virulence (93). For example, a variant with a predilection for replication in the respiratory mucosa rather than the alveoli may cause symptoms like rhinorrhea, nasal congestion, sore throat, and cough. A variant with a predilection for alveoli may cause pneumonia and ARDS. For example, sore throat was a more common symptom during waves predominated by the Omicron variant in comparison with alteration in sense of smell which was relatively more common during waves predominated by the *Delta* variant (92). The omicron variant adopts a different strategy from that of Delta and other variants to adapt to the host. The mutation present in omicron can result in different cell entry pathway therefore this has been shown to have a bearing on the tissue sites preferred for viral replication (94). Omicron variant infection is not enhanced by TMPRSS2 unlike the Delta variant but is largely mediated *via* the endocytic pathway. Also, the Omicron variant shows less efficient replication and fusion activity when compared with the Delta variant in TMPRSS2-expressed cells. The difference in entry pathways between the Omicron and Delta variants may have an implication on the clinical manifestations or disease severity (95). It is also possible that host immunity prevents lower respiratory tract clinical diseases such as pneumonia. A variant may have altered tissue tropism with a predilection for the gastrointestinal tract (93) resulting in gastrointestinal symptoms such as nausea, vomiting, and diarrhea. It has also been reported that different variants may have a different predilection to cause post-COVID-19 sequelae or Long COVID-19 (96). There is evidence that relative to the ancestral strain, the *Alpha* variant was more virulent, and *Delta* was even more virulent than *Alpha* (97, 98). Some studies have shown that the *Omicron* variant was less virulent than past variants (99) and did not replicate as well in alveoli as past variants (100). While countries such as South Africa and India had relatively mild *Omicron* waves, in Hong Kong the Omicron wave was quite lethal (101). The emergence of variants at different time points in populations with differing population immunity from vaccination and/or past infection has resulted in difficulty in conclusively identifying specific mutations as responsible for greater virulence or altered tissue tropism. However, studies in cell culture and animal models have identified specific mutations as likely to cause greater virulence. For example, mutations in *Delta* increase cell-to-cell fusion, syncytia formation, and cytopathic effects in cell cultures and greater pathogenic effects in lungs in animal models (98). It is thought mutations in the S protein at the furin cleavage site may play a critical role in virulence (102, 103). Spike mutations at the RBD and NTD can allow a variant to partially evade neutralizing antibodies and humoral immunity, and this may allow some variants like Omicron to cause more reinfections and breakthrough infections (104). While such mutations are less likely to evade cellular immunity, SARS-CoV-2 does have the inherent ability to downregulate or alter MHC-I expression and may thereby have some impact on cellular immunity (105). It is also possible that viral mutations may alter interferon expression in the host, and thereby impact innate immunity (106). Thus, a variant may have mutations that allow the virus to evade host immune defenses, and this may have an impact on the clinical presentation and severity of infection in a population with immunity. Thus, SARS-CoV-2 variants may have differences in virulence, tissue tropism, and post COVID-19 complications. However, changing population immunity over time has made it difficult to disentangle the impact of host immunity and variant mutations on clinical presentation and severity of SARS-CoV-2 infection.

SARS-CoV-2 VOC can also result in therapeutic dilemmas. As already discussed, some variants may cause more severe disease than others. The *Omicron* variant, which was thought to cause milder disease, required less aggressive intervention and was more likely to be suitable for out of hospital care, whereas the *Delta* variant, which caused more severe disease, carried a greater likelihood of requiring hospital-based care including respiratory support and intensive care. Similarly, anti-viral therapeutics for COVID-19 include drugs like Remdesivir, Favipiravir, Molnupiravir and Paxlovid as well as monoclonal antibody therapies such as Casirivimab-Imdevimab, Sotrovimab, Bamlanivimab-Etesevimab, and Bebtelovimab; and mutations of SARS-CoV-2 variants have been identified during *in vitro* experiments that may confer resistance to Remdesivir by different mechanisms (107). In an interesting case of an immune compromised patient who developed a protracted SARS-CoV-2 infection and was treated with Remdesivir, the E802D mutation in nsp12 RdRp. *In vitro* experiments demonstrated that this mutation allowed greater *in vitro* viral replication in the presence of Remdesivir (108). While this *in vitro* resistance to Remdesivir does not mean there will be a loss of therapeutic efficacy, it should alert us to the possibility that therapeutic strategies will need to change in response to future variants. Remdesivir acts by binding to RdRp and thereby starts its function after adding three nucleotides (109). Hence mutations at the RdRp gene are expected to impair the effectiveness of Remdesivir. Likewise, the effectiveness of monoclonal antibody treatments has been severely impacted by variants of concern (81). Alteration in binding epitope structure of the S protein may abolish binding capacity or reduce binding affinity of antibodies. While Casirivimab-Imdevimab was effective as a treatment in patients infected with the *Delta* variant, it was ineffective against the *Omicron* variant. While Sotrovimab was effective against the *Omicron BA.1*, it was ineffective against *BA.2*. When *Omicron* first emerged, it resulted in the loss of Casirivimab-Imdevimab as a therapeutic option. During the first few weeks of *Omicron's* emergence, identifying *Delta* infections from *Omicron* infections was important as the decision to use monoclonal antibody treatment and which agent to use depended on the variant causing infection. With the emergence of *Omicron BA.2*, Sotrovimab was lost as a therapeutic option. While Bebtelovimab retains effectiveness against currently circulating variants, it seems it is only a matter of time before a variant emerges that impacts the effectiveness of Bebtelovimab. Thus, the variants causing COVID-19 can have an impact on the site of care, the intensity of care and the therapeutic options that will be effective.

## The impact of SARS-CoV-2 variants on transmissibility and immune susceptibility

The transfer of a virus from an infected host to a susceptible host is termed viral transmission. SARS-CoV-2 is a respiratory virus; therefore, its transmission occurs mainly *via* air (110). Two identified primary routes of SARS-CoVo2 transmission include direct transmission occurring *via* droplets or aerosol and indirect transmission happening through fomites (2, 111, 112). Viral transmission is a complex process and involves multiple steps such as viral transfer between hosts, successful attachment of viral particles to its target host cell leading to initiation of infection, and immune status of the host at the time of exposure. The viral-host attachment process involves the successful interaction between host cell receptor ACE2 and viral envelope spike protein *via* RBD and acts as a key determinant for SARS-CoV-2 viral transmission. However, a few recent studies have also reported the ACE2 independent cellular infection of SARS-CoV-2 (113–115), though the detailed mechanism and adaptation of this mode of viral infection is still under investigation. Similarly, the majority of the antibodies against SARS-CoV-2 are generated against the viral spike protein and antigenic changes in this protein have been reported to gain the ability of viral immune escape (116, 117). These factors collectively govern the overall rate of viral transmission and subsequent infection in recovered and vaccinated individuals. SARS-CoV-2 is continuously undergoing mutational and antigenic changes and accumulating these changes in the SARS-CoV-2 genome for better fitness giving rise to SARS-CoV-2 variants, namely *Alpha, Beta, Gamma, Delta*, and *Omicron*. However, in the case of the Omicron variant most of these mutational changes have been detected in the spike encoding region of the genome (118). The antigenic changes in the spike protein of SARS-CoV-2 variants had demonstrated the enhanced transmission capabilities and ability to escape the immune response generated *via* either natural infections or vaccinations. The attribute of enhanced transmission is contributed *via* two mechanisms, i.e., increased affinity of spike RBD for the human ACE2 receptor (hACE2) with a stable conformation of the spike-hACE-2 complex during viral attachment, and easy recognition of the S1/S2 cleavage site by furin proteases leading to activation of the spike protein for efficient viral entry (119).

Likewise, the immune escape ability is gained by accumulating the antigenic changes in the spike encoding region of the viral genome. The details about the mutational changes in each variant and their effect on the viral transmission and immune escape are discussed below.

### *Alpha* VOC

**Figure 5:**
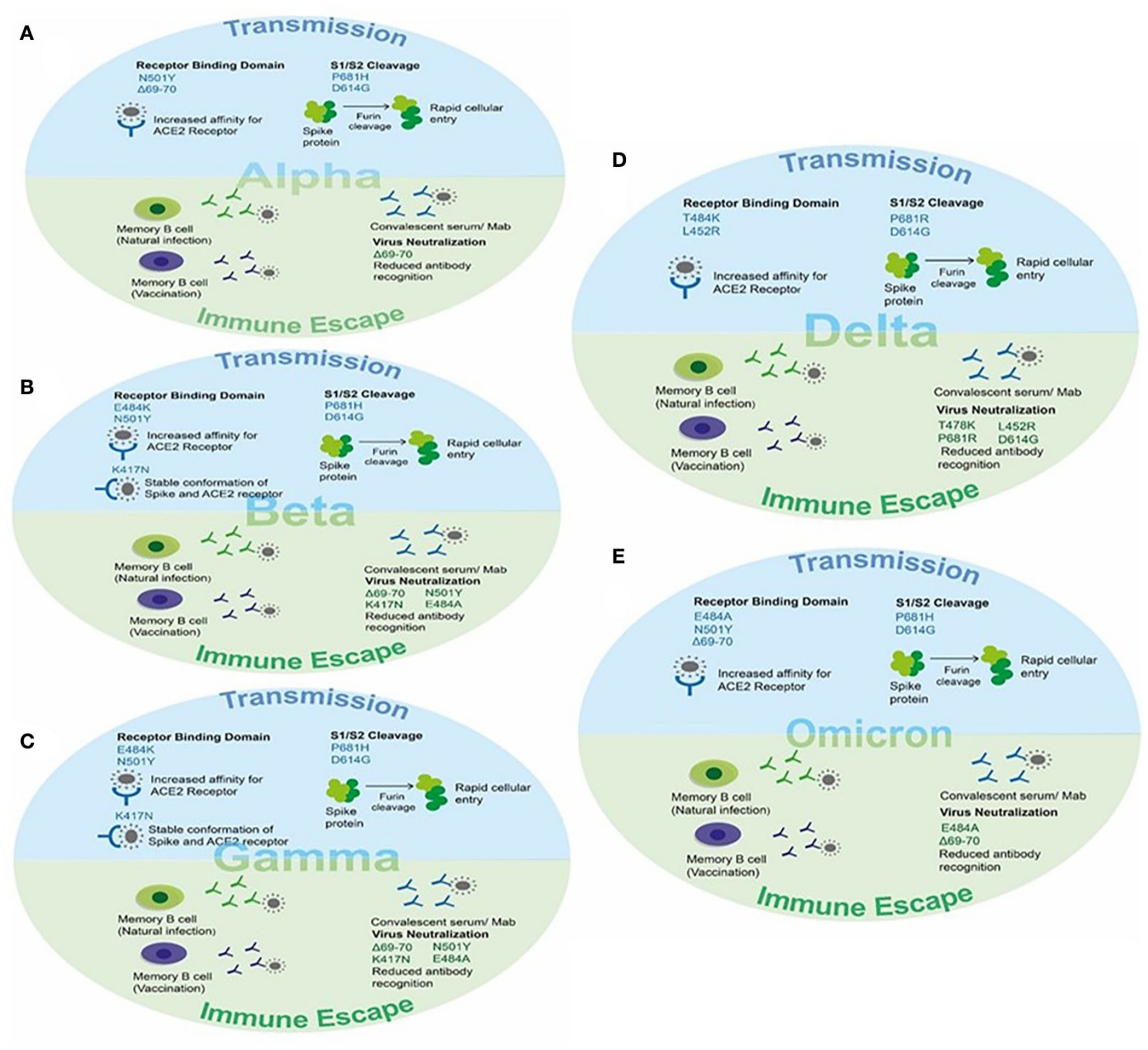
The effect of key mutations of SARS-CoV-2 variants on virus transmission and immune susceptibility. **(A)** Key mutations on *Alpha VOC* involved in transmission and immune escape. **(B)** Key mutations on *Beta VOC* involved in transmission and immune escape. **(C)** Key mutations on *Gamma VOC* involved in transmission and immune escape. **(D)** Key mutations on *Delta VOC* involved in transmission and immune escape. **(E)** Key mutations on *Omicron VOC* involved in transmission and immune escape.

The *Alpha* VOC was reported to be 40–90% (95% CI: 38–130%) more transmissible than the ancestral SARS-CoV-2 strain and could evade the immune response in naturally recovered individuals (32, 47). The higher transmissibility was due to the mutational changes in the spike protein, particularly in the RBD region, and evasion of antibody response was due to the changes in the N-terminal domain (NTD) of the spike protein. The N501Y mutation, present in the receptor-binding motif of the spike, serves as the key contact residue between the spike and its receptor human ACE-2, therefore, reported to increase the affinity of RBD for hACE-2 receptors leading to rapid transmission between hosts (50, 120). The P681H mutation present near the S1/S2 cleavage site resulted in rapid conversion of the spike to active S1/S2 by furin proteases, thus increasing the rate of virus transmission and promoting viral entry (121, 122). The 69–70 deletion in NTD of spike protein demonstrated increased viral infectivity *via* an increase in the incorporation of cleaved S2 in viral spike and rapid syncytium formation (123, 124). Moreover, 69–70 deletion, in combination with other spike mutations such as D796H, could provide immune escape (123, 124), and antibodies generated after recovery or *via* vaccination showed decreased susceptibility against double mutants possibly causing infection in vaccinated individuals and re-infections in recovered individuals (125–127). This deletion in NTD has also been reported to decrease the recognition of this region by NTD specific monoclonal antibodies (128). Additionally, the D614G mutation has been reported to possess increased viral fitness and viral titer in the *in-vitro* system (129, 130). The enhanced infection was suggested because of reduced S1 shedding and increased incorporation of the spike protein into the virion (131, 132) (Figure 5A).

### *Beta* VOC

The *Beta* VOC was reported to be more transmissible than the ancestral SARS-CoV-2 strain and successfully evaded the immune response in vaccinated individuals reducing the efficacy of vaccines. Two mutations, namely E484K and N501Y, are present within the receptor-binding motif of the spike and hence increase the affinity of RBD for ACE-2 resulting in rapid viral attachment (50, 133, 134). The K417N was detected in RBD and contributes to the stable conformation of spike-ACE-2 providing the stable interaction by giving more negative free energy. In addition to this, the K417N, E484K, and N501Y mutations were able to abolish the binding of generated antibodies against the spike protein (135). Therefore, the vaccine efficacy was found to be significantly reduced in cases of infection and disease symptoms with the *Beta* variant (Figure 5B).

### *Gamma* VOC

The *Gamma* VOC was reported with 17 amino acid substitutions, 10 of which lie in the spike region. The three spike mutations K417T, E484K, and N501Y were of particular interest and contributed to increased pathogenesis (52). Two of these mutations, namely E484K and N501Y, are within the receptor-binding motif of the spike and hence increase the affinity of RBD for ACE-2 resulting in rapid viral attachment (50, 53, 136). The K417T was present in RBD and contributed to the stable conformation of spike-ACE-2 providing the stable interaction by giving more negative free energy (116). In addition to this, the K417T and N501Y mutations were able to abolish the binding of generated antibodies against the spike protein (137). Therefore, the vaccine efficacy was found to be significantly reduced in cases of infection with the *Beta* variant (Figure 5C).

### *Delta* VOC

The four spike mutations of the *Delta* VOC, i.e., T478K, P681R, L452R, and D614G, were of particular interest and contributed to increased transmission and immune escape against a generated immune response. Two of these mutations, namely T484K and L452R are within the receptor-binding motif of the spike and hence increase the affinity of RBD for ACE-2 resulting in rapid viral attachment (54–56). The D614G mutation present in the SD2 domain and P681R mutation present in the S1/S2 cleavage site assists in rapid conversion of the spike to active S1/S2 by furin proteases resulting in increased viral entry. In addition to this, the D614G and other mutations were able to abolish the binding of generated antibodies against the spike protein (116). Therefore, the vaccine efficacy was found to be significantly reduced in cases of infection and symptomatic disease with the *Delta* variant (Figure 5D).

### *Omicron* VOC

This variant reported to have 60 mutations as compared to the ancestral strain. Among them, 32 mutations were present in the spike protein affecting the viral transmission and immune response in vaccinated individuals as well as recovered patients. The main mutations present in RBD of the spike protein are K417N, T478K, E484A, and N501Y affecting the binding of the spike protein for hACE-2 *via* increased affinity, stable S-ACE-2 interaction, and more negative free energy as reported in other variants (50, 52, 54, 135). The 69–70 deletion in NTD also present in the *Alpha* variant contributed to the rapid transmission and evading of the antibody immune response (124). The P681H and D614G mutations present near the S1/S2 furin cleavage site resulted in an increased rate of virus transmission (121, 122, 129, 130). The other reported mutation and their effect on viral pathogenesis are still underway (Figure 5E).

## Conclusion

COVID-19 has changed the way the world looks at infectious diseases in general and viral diseases in particular. Knowledge and technology transfer of information regarding disease presentation, disease management, development of therapeutics, and surveillance strategies has seen an unprecedented improvement which can be mainly attributed to a joining of forces of the public health professionals, paramedics, law enforcement, researchers, policy makers, and hospital infrastructure to fight the pandemic. Lessons learned have been many; and the learning curve has been quite steep.

One of the first lessons learned is the significance of transparency of disease reporting at the global level. Had the first report of pneumonia-like disease been reported in a more transparent manner, would the disease have been contained better and not have reached a pandemic level? Is the policy in place now, at the global level, to address this issue if another similar outbreak strikes in the future? The next crucial lesson learned is the importance of a country's medical services and their preparedness. This pandemic revealed that whether a country was developed, developing or underdeveloped, preparedness during an emergency can save lives. The impact of the first wave in terms of number of deaths and hospitalization in the Western world vis-à-vis developing countries such as India is a classic example.

Another important lesson learned is the importance of molecular surveillance. If not for the extensive whole genome sequencing and the robust analyses tools, we would not have identified the variants so early and thereby understood some of the details of the varied disease presentations both at the population level and at the individual level. Identification of variants has resulted in providing both personalized therapy and community-level disease management with equal proficiency. Availability of these sequences has ensured development of faster diagnostics with increased specificity and sensitivity in record time thereby aiding in disease management. Other peripheral yet important attributes of whole genome sequencing have been the mushrooming of these facilities even in tertiary health centers and smaller research facilities coupled by the growth of a data analysis framework, which is being put into use for other infectious diseases and pathogens. Several countries now have developed centralized data management systems making data availability much more streamlined.

The pandemic taught us the price of complacency; the Delta wave in India is one such costly example. And India learned well in the process. The vaccination drive was implemented with renewed rigor; use of traditional alternative medicines was revived and used extensively to combat the disease. Dogmas were rewritten in vaccine development and novel strategies developed in therapeutics. Knowledge from every field was adopted, adapted, and tested to understand and curb the pandemic with varied success. The most important message conveyed in the last 2 years of the pandemic has been the public health awareness imbibed by the general population regarding disease, genome sequencing, personal protection, preparedness, virology, and human immunology. While the deadly Ebola and the Nipah virus outbreaks that occurred in the recent past were lessons to the experts, COVID-19 has turned out to be a crash course to the uninitiated in several varied aspects.

How can we best benefit from all the lessons learned in the pandemic? Expanding and diversifying the experience and the expertise acquired from the pandemic to other health concerns will be a significant consequence. Sewage water genome surveillance, ultra-high throughput next generation sequencing platforms, humungous real-time data management systems, multi-level data analyses pipelines, novel diagnostic POCT strategies, and new vaccine development approaches have been put in place since 2020. It is up to the community to utilize these procedures to prevent, predict, monitor, control, and manage emerging viral diseases.

## Author contributions

SS and SM conceptualized the study and compiled the complete manuscript with inputs from all authors. SC and SM contributed to SARS-CoV-2 molecular evolution, SARS-CoV-2 mutations, and variants. SCS, BK, SA, VC, NV, and JS contributed to impact of SARS-CoV-2 variants on Diagnostics/Detection. SP and JS contributed to impact of SARS-CoV-2 variants on clinical presentation and treatment. DV and DM contributed to impact of SARS-CoV-2 variants on transmissibility and immune susceptibility. SS, SC, DV, and SM contributed to the implication of emerging SARS-CoV-2 variants of concern. SS and JS provided all resources and supervised the overall study. All authors contributed to the article and approved the submitted version.

## Funding

ICGEB core funds were utilized for the preparation of this review.

## Conflict of interest

The authors declare that the research was conducted in the absence of any commercial or financial relationships that could be construed as a potential conflict of interest.

## Publisher's note

All claims expressed in this article are solely those of the authors and do not necessarily represent those of their affiliated organizations, or those of the publisher, the editors and the reviewers. Any product that may be evaluated in this article, or claim that may be made by its manufacturer, is not guaranteed or endorsed by the publisher.
